# Selection of reference genes for expression studies with fish myogenic cell cultures

**DOI:** 10.1186/1471-2199-10-80

**Published:** 2009-08-10

**Authors:** Neil I Bower, Ian A Johnston

**Affiliations:** 1 Scottish Oceans Institute, School of Biology, University of St Andrews, St Andrews, Fife, KY16 8LB, UK

## Abstract

**Background:**

Relatively few studies have used cell culture systems to investigate gene expression and the regulation of myogenesis in fish. To produce robust data from quantitative real-time PCR mRNA levels need to be normalised using internal reference genes which have stable expression across all experimental samples. We have investigated the expression of eight candidate genes to identify suitable reference genes for use in primary myogenic cell cultures from Atlantic salmon (*Salmo salar *L.). The software analysis packages geNorm, Normfinder and Best keeper were used to rank genes according to their stability across 42 samples during the course of myogenic differentiation.

**Results:**

Initial results showed several of the candidate genes exhibited stable expression throughout myogenic culture while *Sdha *was identified as the least stable gene. Further analysis with geNorm, Normfinder and Bestkeeper identified *Ef1α*, *Hprt1*, *Ppia *and *RNApolII *as stably expressed. Comparison of data normalised with the geometric average obtained from combinations of any three of these genes showed no significant differences, indicating that any combination of these genes is valid.

**Conclusion:**

The geometric average of any three of *Hprt1*, *Ef1α*, *Ppia *and *RNApolII *is suitable for normalisation of gene expression data in primary myogenic cultures from Atlantic salmon.

## Background

Skeletal muscle myogenesis involves numerous steps including the proliferation, migration and fusion of myoblasts to form myotubes; the onset of myofibrillargenesis, and the maturation and hypertrophy of muscle fibres [1,2]. Myogenesis in teleost fish has several unique features compared to mammals, including the production of myotubes throughout much of adult life [3]. The *in vitro *culture of fish myogenic cells is an attractive system for studying the formation and differentiation of myotubes and examining the effects of various regulatory molecules on gene expression under precisely controlled conditions [4,5]. Furthermore, since traditional gene "knockouts" are unavailable in fish, cell culture provides a viable alternative for functional assays.

A pre-requisite for the quantitative measurement of gene expression is the identification of suitable reference genes to normalise the data [6,7]. Reference genes are required to normalise for differences in RNA input and mRNA/rRNA ratios between samples [8]. Also, differences in reverse transcription efficiencies between samples can occur due to the presence of inhibitors carried over from the RNA purification [8], and the presence of PCR inhibitors can affect the number of cycles required to reach the quantification cycle value [9]. As gene expression patterns change in response to many stimuli, stable expression of reference genes needs to be confirmed for each experimental system. For example, genes identified as being stable in whole muscle samples, may not be suitable as reference genes in myogenic cell culture due to the vast changes in cell metabolism and structure that occur during the transition from myoblast to myotube. Previous myogenic cell culture experiments using the C2C12 cell line have relied on *Actb *[10] and *Gapd *[11] as internal reference genes, however, the validity of these genes is questionable as *Gapd *[12,13] and *Actb *[14] expression has been shown to vary considerably.

A number of computer based analysis packages have been developed which analyse gene expression patterns and allow for the identification of stable reference genes. Vandesomple et al [15] designed the widely used geNorm package which uses a pairwise analysis of gene expression to identify stable reference genes. Likewise, Bestkeeper [16] performs a pairwise comparison, whereas Normfinder [17] uses a mathematical model to estimate overall expression variation of candidate reference genes, but also the variation between sample groups. Vandesomple et al [15] demonstrated that use of a single reference gene can lead to aberrant gene expression values, and now it is widely accepted that using several reference genes for normalisation is preferable.

Currently there is no information available on reference gene stability in fish myogenic cell cultures. In this paper we examine the stability of eight potential reference genes during the transition from single nucleated myoblasts to multinucleated myotubes in myogenic cell cultures derived from Atlantic salmon, one of the most commercially important aquaculture species.

## Results

**Figure 1:**
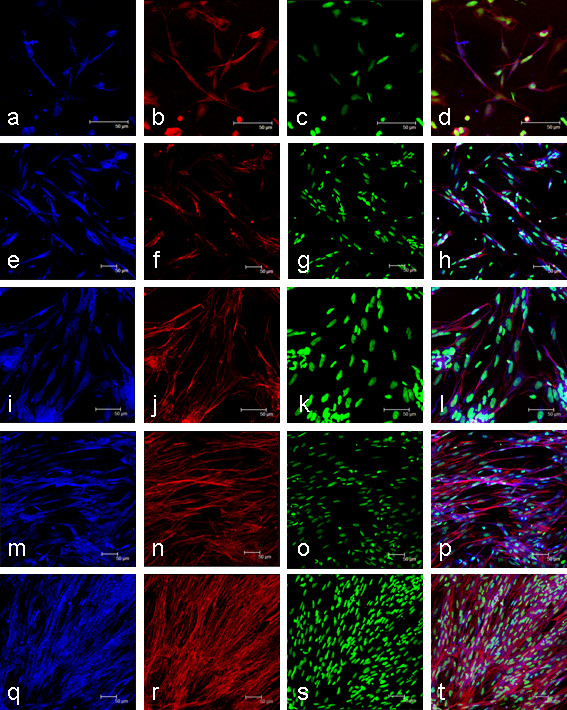
**Growth and differentiation of myogenic cells extracted from *Salmo salar *fast myotomal muscle**. Growth is shown at 2 d (a-d), 5 d (e-h), 8 d (i-l), 11 d (m-p) and 14 d (q-t) after cell extraction. Myogenic cells were identified by positive desmin staining (a,e,i,m,q). Actin counterstained with phalloidin (b,f,j,n,r) and nuclei stained with sytox green (c,g,k,o,s) also confirmed the presence of multinucleated myotubes shown in the overlay (d,h,l,p,t). Scale bars represent 50 μm.

Cell cultures were visualised using confocal microscopy and the phenotype of cells determined at 2 d, 5 d, 8 d, 11 d and 14 d (Figure 1). The myogenic nature of the cell culture was confirmed by the presence of the myogenic marker desmin (Figure 1 a, e, i, m, q) and the presence of multi-nucleated myotubes visualised by Alexa Fluor 568-phalloidin stained actin filaments (Figure 1 b, f, j, n, r) and nuclei stained with sytox green (Figure 1 c, g, k, o, s). At 2 d, all cells were mononucleic (Figure 1a–d), which then fused to form small myotubes at 5 d (Figure 1e–h) and 8 d (Figure 1i–l) and then as the culture progressed, large myotubes (Figure 1m–p) and sheets of large multi-nucleated myotubes at 14 d (Figure 1q–t).

Each of the candidate reference genes tested gave amplification from cDNA derived at each time point of the cell culture, while the no template control (NTC) and minus reverse transcription controls (-RT) gave no signal. The specificity of each primer was by confirmed by the presence of a single band on agarose gel electrophoresis and the presence of a single peak in the dissociation curve analysis which exactly matched the dissociation curve of a plasmid standard of known sequence. Amplification of the correct product was confirmed in each case through the sequence analysis of cloned PCR products.

### Reference gene stability

**Figure 2:**
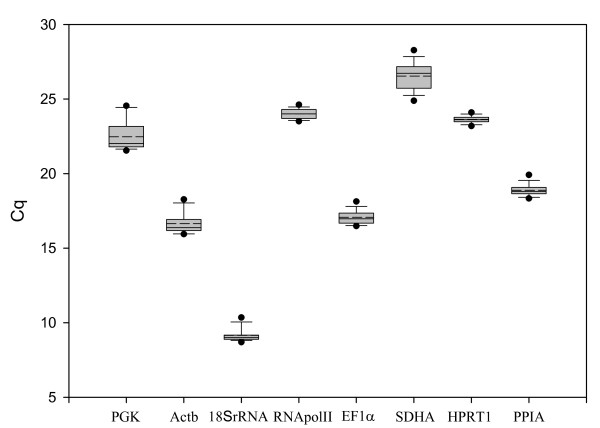
**Expression values for all genes at all time points from Atlantic salmon myogenic cell culture**. The raw quantification cycle (Cq) values (n = 42) are represented by box and whisker diagram (box represents quartiles). The mean value is indicated by the dashed line and the 5^th ^and 95^th ^percentiles are indicated by the dots above and below each plot.

**Figure 3:**
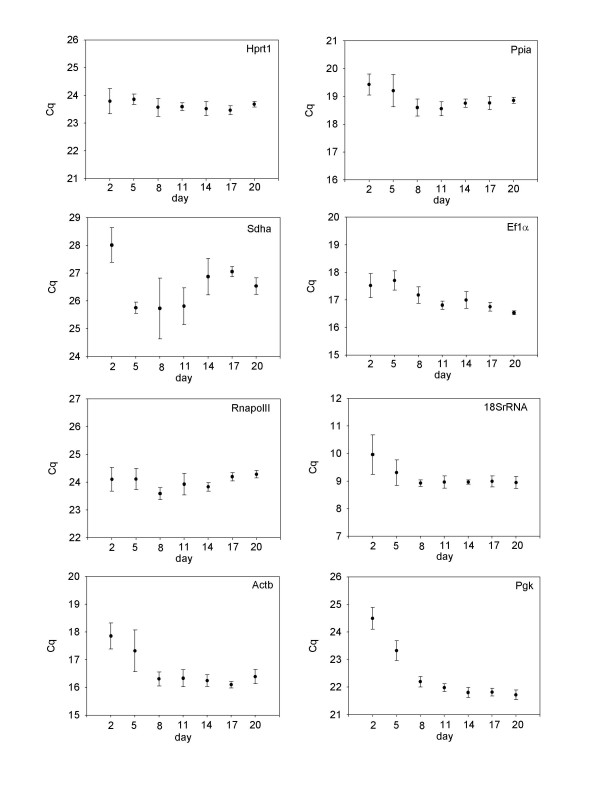
**Individual expression profiles for each candidate reference gene at each day of culture**. Values shown are raw Cq values represented as mean ± SD (n = 6).

The cell culture undergoes many structural and metabolic changes during the transition from mononucleic cells to multi-nucleated myotubes. We therefore chose to analyse the expression of genes from early time points as well as time points after the culture has produced multi-nucleated myotubes (17 d and 20 d). The analysis of reference gene stability can thus be performed in three phases, the first in developing myotubes (2 d – 11 d), the second in established myotubes (11 d – 20 d) and the third covering all time points. Based on the raw expression data (Figure 2), *Sdha *and *Pgk *show higher levels of variance than the remaining genes and appear the least stable. Figure 3 shows the raw expression values obtained for each gene at each of the time points sampled. For several of the genes, there is higher intergroup variation indicating that these genes are differentially regulated during the progression of the cell culture. For example, *18SrRNA*, *Pgk*, and *Actb *all have higher Cq values at 2 d and 5 d when the culture is predominantly mononucleic cells than they do at later time points when myotubes have formed. *Sdha *has high inter-assay and intra-assay variation and is clearly unsuitable as a reference gene in Atlantic salmon myogenic culture.

As inspection of raw Cq values alone is insufficient for determining gene expression stability, the data obtained were further analysed using three software packages Bestkeeper, geNorm and Normfinder. Each package uses a different algorithm to determine the most stable reference gene, and as no single method has been accepted as the most appropriate for identifying stable gene expression, all three packages were used for analysis.

### geNorm Analysis

**Figure 4:**
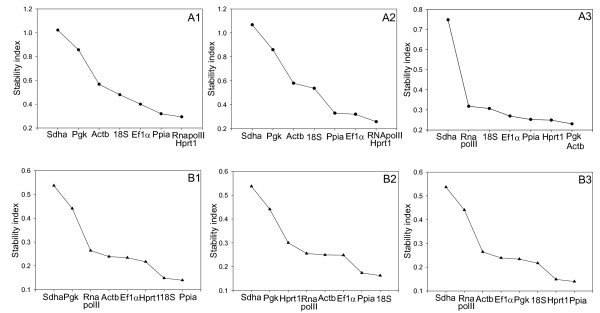
**Stability indices calculated with geNorm (A) and Normfinder (B)**. Stability indices are shown for all time points (1), developing myotubes at days 2–11 (2) and in established myotubes at days 11–20 (3). Stability of gene expression is inversely proportional to the stability index, so least stable genes are to the left and the most stable to the right for each graph.

Data analysis using geNorm was performed two ways. The first method used the absolute values derived from a plasmid standard curve as input, the second used the delta Cq method, with the PCR efficiencies based on a dilution series of pooled cDNA samples. The results from the three geNorm analyses covering all time points (absolute value method), developing myotubes and established myotubes are shown in figure 4. When all samples were analysed, the genes were ranked in an identical order using both analysis methods from most to least stable: *Hprt1*>*RNApolII*>*Ppia*>*Ef1α*>*18SrRNA*>*Actb*>*Pgk*>*Sdha*. Analysis of days 2–11 using both analysis methods revealed the same order of stability as when all days were analysed except for *Ef1 *and *Ppia *swapping order. When days 11–20 are analysed using the absolute method, the order changes from most to least stable: *Pgk*>*Actb*>*Hprt1*>*Ppia*>*Ef1*>*18SrRNA*>*RNApolII*>*Sdha*. When the delta Cq method was used, the order changed to:*Pgk*>*Hprt1> Actb*>*Ppia*>*Ef1*>*18SrRNA*>*RNApolII*>*Sdha*. The change in order from days 2–11 to days 11–20 likely reflects the changes in metabolism and structure that occur during differentiation and growth process as myotubes form. In all three analyses, the M values obtained for *Hprt1*, *Ppia *and *Ef1α *were quite similar, ranging from 0.23–0.36. Based on the similar M values, it would appear that any combination of *Hprt1*, *Ppia *and *Ef1α *would be suitable for normalisation.

### Normfinder Analysis

The stability of candidate reference genes was also analysed using Normfinder (Figure 4). The overall rank of genes from most to least stable for all time points was: *Ppia*>*18SrRNA*>*Hprt1*>*Ef1α*>*Actb*>*RNApolII*>*Pgk*>*Sdha*. In developing myotubes the genes were ranked: *18SrRNA*>*Ppia*>*Ef1α*>*Actb*>*RNApolII*>*Hprt1*>*Pgk*>*Sdha*, and in established myotubes: *Ppia*>*Hprt1*>*18SrRNA*>*Pgk*>*Ef1α*>*Actb*>*RNApolII*>*Sdha*. It is noteworthy that with the exception of *Sdha *and *Pgk*, all genes had stable expression in all three analyses, with stability indices between 0.3 and 0.08.

### Bestkeeper Analysis

Using the initial statistics produced by Bestkeeper (Figure 2), the genes were ranked in the following order from most to least stable: *Hprt1*>*RNApolII*>*Ppia*>*Ef1α*>*Sdha*>*Actb*>*18SrRNA*>*Pgk *when all time points were examined. For 2d-11d, the genes were ranked: *Hprt1*>*RNApolII*>*Ef1α*>*Ppia*>*Actb*>*18SrRNA*>*Sdha*>*Pgk *and for 11d-20d: *Pgk*>*Hprt1*>*Ppia*>*RNApolII*> *Ef1α*>*Actb*>*18SrRNA*>*Sdha*. All candidate reference genes examined were positively correlated with each other (Table 1), with the highest correlations found between *Actb*/*Pgk *(r = 0.914) and *Ppia*/*Actb *(r = 0.874). Correlations between the remaining genes ranged from 0.198 for *RNApolII*/*Ef1α *to 0.814 for *18SrRNA*/*Actb *(Table 1). The low level of correlation between many of the genes is due to the small inter and intra-group variation observed for the majority of the genes. From the initial statistics, the four least stable genes were removed from further analysis. The algorithm used in Bestkeeper then calculates the correlation of each gene with the Bestkeeper Index which is calculated as the geometric mean of the candidate reference genes. The candidate reference genes were ranked in order based on their correlation with the Bestkeeper index based on the four most stable genes. For all time points the genes were ranked in order most to least stable: *Ppia*>*Ef1α*>*Hprt1*>*RNApolII *whereas in developing myotubes the order was: *Ppia*>*Ef1*>*RNApolII*>*Hprt1 *and in established myotubes the ranking was *Ef1α*>*Ppia*>*Pgk*>*Hprt1*.

**Table 1:** Correlations between candidate reference genes expression patterns

	*Pgk*	*Actb*	*18SrRNA*	RNApol II	*EF1α*	*Sdha*	*Hprt1*	*Ppia*
*Actb*	0.914	-	-	-	-	-	-	-
*18SrRNA*	0.755	0.814	-	-	-	-	-	-
*RNApolII*	0.229*	0.449	0.382	-	-	-	-	-
*EF1α*	0.779	0.788	0.603	0.198*	-	-	-	-
*Sdha*	0.393	0.343	0.517	0.325	0.076*	-	-	-
*Hprt1*	0.514	0.612	0.488	0.57	0.656	0.281*	-	-
*Ppia*	0.732	0.874	0.672	0.664	0.705	0.372	0.64	-

Pearson correlation coefficients (r) based on the quantification cycle (Cq) value between reference genes. Probability greater than 0.05 is indicated by an asterisk.

### Normalisation

**Figure 5:**
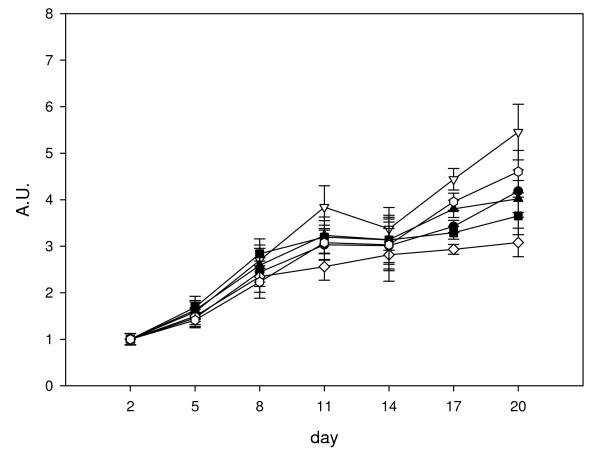
**Normalisation of desmin mRNA expression to various combinations of the geometric average for two genes**. Data shown are all calculated relative to day 2, so that day 2 values are equal to 1 arbitrary unit (A.U.). Six normalisation factors were derived by calculating the geometric averages of the following gene combinations: A: *Hprt1, Ppia *(closed circle); B: *RNApolII, Hprt1 *(open triangle); C: *EF1α*, *Hprt1 *(closed square); D: *EF1α, Ppia *(open diamond); E: *RNApolII*, *EF1α*(closed triangle); F: *RNApolII*, *Ppia *(open circle). Values shown are the mean normalised value ± S.E. (n = 6).

**Figure 6:**
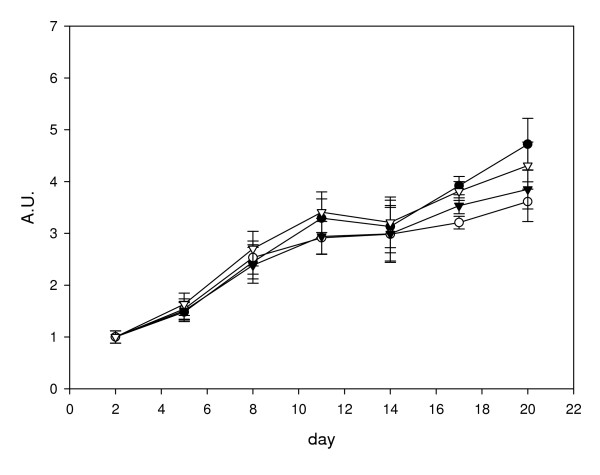
**Normalisation of desmin mRNA expression to various combinations of the geometric average of three genes**. Data shown are all calculated relative to day 2, so that day 2 values are equal to 1 arbitrary unit (A.U.). Four normalisation factors were derived by calculating the geometric averages of the following gene combinations: A: *EF1α*, *RNApolII*, *Hprt1 *(closed circle); B: *EF1α*, *Ppia*, *Hprt1 *(open circle); C: *EF1α*, *RNApolII*, *Ppia *(closed triangle); D: *Ppia*, *Hprt1*, *RNApolII *(open triangle). Values shown are the mean normalised value ± S.E. (n = 6).

Based on the results from the three analysis methods, four genes, *EF1α*, *Ppia*, *Hprt1 *and *RNApolII *are consistently stable. In order to assess the stability of the normalisation factors obtained, we first compared the normalised expression of *Des *to various combinations of the geometric average of two genes (Figure 5). Six normalisation factors were derived by calculating the geometric averages of the following gene combinations: A: *HPRT1, PPIA*; B: *RNApolII, HPRT1*; C: *EF1α*, *HPRT1*; D: *EF1α, Ppia*; E: RNApolII, *EF1α*; F: *RNApolII*, *Ppia*. We found that there were significant differences (ANOVA P < 0.05) in *Des *gene expression at day 11 (B v D), at day 17 (A v B, D, and F; B v C, D and E; C v E and F; D v E and F) and at day 20 (B v C, D and E; D v F). We therefore examined normalisation using geometric average of three genes (Figure 6). Four normalisation factors were derived by calculating the geometric averages of the following gene combinations: A: *EF1α*, *RNApolII*, *Hprt1*; B: *EF1α*, *Ppia*, *Hprt1*; C: *EF1α*, *RNApolII*, *Ppia*; D: *Ppia*, *Hprt1*, *RNApolII*. No significant differences were observed (ANOVA p = 0.05) when the normalised data at each time point were compared between the different normalisation factors, indicating that using the geometric average of any three of these genes is suitable for normalisation.

## Discussion

In this study, we examined the expression of eight candidate reference genes for normalisation of quantitative real-time PCR data from a primary culture of Atlantic salmon myogenic cells. The identification of genes with stable expression in all samples of an experiment is crucial as it is necessary to normalise for variability between samples introduced during the production of the cDNA [6,7]. As a universal reference gene with stable expression in all experimental systems is not available, suitable reference genes for each experiment need to be determined.

Myogenic cell culture is characterised by distinct phases where cells first proliferate, and then fuse to form multinucleated myotubes [2]. We therefore identified genes that were stable early time points where the majority of the cells are mononucleic and forming small myotubes (culture days, 2–11) those stable in established myotubes (days 11–20) and those most stable for the entire culture period. When the raw Cq values obtained at each time point are compared (Figure 3), it is clear that for the majority of the genes examined, stable expression is observed once myotubes have become established in the culture, whereas higher intra and intergroup variation is observed when myotubes are developing. For example, *Pgk*, a glycolytic enzyme, appears to be upregulated as myotubes start to form, and then has stable expression in established myotubes as indicated by the low inter and intra-group variation (Figure 3).

To identify stably expressed genes, analysis packages such as geNorm and Bestkeeper perform a pairwise comparison of gene expression across the various samples in an experiment. Therefore it is crucial that genes used are not co-regulated or present on the same pathway, as co-regulated genes will likely have similar expression patterns and would therefore appear to be stably expressed in any biological experiment. For this reason we chose genes involved in a number of different biological processes (Table 2), such as nucleotide recycling (*Hprt1*), peptide isomerisation (*Ppia*), glycolysis (*Pgk*), citric acid cycle (*Sdha*), ribosome assembly (*18SrRNA*), transcription (*RNApolII*), translation (*Ef1α*) and cytoskeleton structure (*Actb*).

**Table 2:** GenBank accession numbers and function of selected reference gene candidates

Gene symbol	Accession number	Gene Name	Function
*EF1α*	BG933853	Eukaryotic elongation factor 1a	Translation
*RNApolII*	BG936649	RNA polymerase 2	Transcription
*18SrRNA*	AJ427629	18S ribosomal RNA	Component of ribosome
*Ppia*	DY727143	Prolylpeptidyl isomerase A	Peptide isomerisation
*Pgk*	DW536646	Phosphoglycerate kinase	Glycolysis
*Actb*	G933897	Beta actin	Cytoskeleton
*Hprt1*	EG866745	Hypoxanthine phosphoribosyl transferase 1	Purine salvaging
*Sdha*	GE769149	Succinate dehydrogenase complex subunit A flavoprotein	Oxidation of succinate

Based on the M values obtained in geNorm (Figure 4), the stability index from Normfinder (Figure 4) and the descriptive statistics produced by Bestkeeper (Figure 2), it would appear that several of the genes used in this study are suitable for normalisation of gene expression data from salmon myogenic cell cultures. For example, Pfaffl et al [16] recommends using genes that have a standard deviation for the Cq values less than one for calculating a Bestkeeper index. In our study, all genes examined had standard deviation less than one. This is also reflected in the slight changes in the order of gene stability obtained from each of the three software packages. The least stable gene identified by all analysis methods was *Sdha*. *Sdha *has been used as a reference gene in a number of studies using different tissues [18,19], however its high inter and intra-group variation make it unsuitable for normalisation in salmon myogenic cell cultures.

Results obtained from geNorm identify *Hprt1 *and *RNApolII *as the most stable genes when all time points were examined, however, the M values obtained for *Ppia *and *Ef1α *are quite similar and thus these genes are also likely to be suitable for normalisation. The same set of genes was found to be stable in developing myotubes, but differed in established myotubes where *Pgk*/*Actb *were found to be the most stable, although the *Hprt1 *and *Ppia *also had low M values and can be considered stably expressed.

The most stable genes identified for all time points by Normfinder ranked in descending order were *Ppia*>*18SrRNA*>*Hprt1*>*Ef1α*. Both *Ppia *and *Hprt1 *have been reported to give stable expression in mouse C2C12 myotubes [20,21] and *Ef1α *has been reported to have stable expression in some Salmon tissues [22]. As the 18S and 28S ribosomal RNAs are highly abundant and account for the vast majority of RNA, it is unsurprising that *18SrRNA *is found to be stable across the samples as equal amounts of RNA were reverse transcribed. However, Vandesomple et al [15] criticise the use of *18SrRNA *as a housekeeping gene due to its high abundance making baseline subtraction difficult. Also, transcription of rRNA and mRNA occur via RNA polymerase I and II respectively which may lead to imbalances in the two mRNA fractions as reported by Solanas et al [23]. The similar stability indices obtained for *Ppia*, *Hprt1 *and *Ef1α*, identify all of these genes as suitable for normalisation.

Similar to the results of geNorm and Normfinder, Bestkeeper analysis revealed the most stable genes to be *Ppia*, *Ef1α*, *Hprt1 *and *RNApolII*. Interestingly, *Actb*, which has been used as a reference gene in numerous studies [10,24], was found to be the third least stable gene in this analysis, having high intra-group variation in developing myotubes as well as high inter-group variation when comparing developing and established myotubes. These differences in Cq values between developing and established myotubes indicate that *Actb *is differentially regulated during differentiation of Atlantic salmon myogenic cells, as reported in chicken and mouse myoblast culture [25,20] and is therefore unsuitable as reference gene for myogenic culture. Interestingly, *RNApolII *and *Hprt1*, which were identified as the most stable genes in geNorm (Figure 4) had a correlation coefficient of only 0.57, which was lower then for many of the other genes (Table 1). The selection as the most stable genes in geNorm is likely a reflection of the low intra and inter-group variation observed for both of these genes (Figure 3).

Vandesomple et al [15] recommend using the geometric average of three reference genes for accurate normalisation. To assess the suitability of the reference gene candidates, we first normalised the expression of *Des *to combinations of the geometric average of two reference genes from *Ppia*, *Hprt1*, *Ef1α *and *RNApolII *(Figure 5). We found significant differences in *Des *expression at days 11, 17 and 20 when comparing results from different combinations of reference genes. However, when three genes were used, there were no significant differences between any of the combinations of reference genes (Figure 6) indicating that all four genes are suitable for normalisation when the geometric average of three genes is used.

## Conclusion

To the best of our knowledge, this is the first study examining gene expression stability in myogenic culture of a teleost species and thus provides a useful platform for gene expression studies using this system. The data provided in this paper may also be useful in guiding researchers performing myogenic cell culture in other teleost species. We recommend using a three gene normalisation factor using the geometric average of any combination of *EF1α*, *Ppia*, *RNApolII *and *Hprt1*.

## Methods

### Isolation of myogenic satellite cells

Myosatellite cells were isolated using a method similar to that described by Koumans et al [26]. Juvenile Atlantic salmon (*Salmo salar *L) 30 ± 6 g (mean ± s.d., N = 10) were used for each culture. As the experimental animals had not undergone gonadal development, the gender of the fish was not determined. Fast myotomal muscle was dissected under sterile conditions and placed in extraction media consisting of Dulbecco's modified eagle's media (DMEM) 9 mM NaHCO3, 20 mM HEPES (pH 7.4) with 15% (v/v) horse serum and 1 × antibiotics (100 units/ml penicillin G, 100 μg/ml streptomycin sulfate, 0.25 μg/ml amphotericin B) (Sigma, Gillingham, Dorset, UK) at a ratio of 1 gram of muscle per 5 ml extraction media. The tissue was then minced with a sterile scalpel before centrifugation at 300 g for 5 min, and two washes with DMEM without horse serum. The muscle pieces were digested with collagenase (0.2% m/v in DMEM, Type 1a, Sigma, Gillingham, Dorset, UK) for 70 minutes at room temperature in the dark, before centrifugation at 300 g for 5 minutes. The resulting pellet was washed twice with DMEM before being passed through a pipette repeatedly to separate cells.

Samples were further digested with trypsin (0.1% in DMEM) for 20 minutes at room temperature. The resulting cell suspension was centrifuged (300 g, 1 min). The supernatant was poured into 20 × vol of extraction media containing serum to inhibit trypsin activity. The pellet was further digested by a second treatment with trypsin for 20 min at room temperature, before centrifugation at 300 g 1 min. The supernatant was poured into 20 × volume of extraction media. The extraction media containing the cell suspension was centrifuged 300 g, 20 min. Cell pellets were re-suspended in 30 ml of basal medium before mechanical trituration through 10 ml and 5 ml pipettes until cells are separated. The cell suspension was then passed through 100 μm and 40 μm nylon cell strainers (BD Biosciences San Jose, CA, USA) and centrifuged 20 min 300 g. The cells were resuspended in basal media, cell number determined using hymaecytometer, and then diluted to give approximately 1.5 × 10^6 ^cells/ml.

### Cell culture

All cell culture methods were performed using Aseptic technique in a Microflow 2 Advanced biosafety cabinet (Bioquell Ltd, Andover, UK). 6 well cell culture plates (Greiner Bio-One Ltd, Stroudwater, UK) were treated with a 100 ug/ml poly-lysine solution (Sigma, Gillingham, Dorset, UK) at 4 μg/cm^2 ^for 5 minutes at room temperature, then aspirated before 2 washes with sterile water and allowed to air dry. 1 ml of laminin (Sigma, Gillingham, Dorset, UK) in DMEM at 20 μg/ml was applied to each well and incubated at 18°C overnight prior to plated cells. Cell culture was performed using complete medium (DMEM, 9 mM NaHCO3, 20 mM HEPES (pH 7.4), supplemented with 10% foetal calf serum (Sigma, Gillingham, Dorset, UK) and 1 × antibiotics (Sigma, Gillingham, Dorset, UK) which was changed daily.

### Immunofluorescence of culture cells

Cells were grown on glass coverslips treated with poly-L-lysine and laminin as described above. Samples were washed 2 × in PBS, fixed in 4% (m/v) paraformaldehyde for 20 min at room temperature, washed 2 × 5 mins in PBS, permeabilised with 0.2% triton X-100 PBS for 5 minutes, washed 2 × in PBS and then blocked in 5% NGS, 1.5% BSA, 0.1% triton X-100 PBS for 1 hour at room temperature. All antibody steps were performed in PBST (1% BSA, 0.1% triton X-100 in PBS). Desmin antibody (Sigma, Gillingham, Dorset, UK) was diluted 1:20 in PBST and incubated overnight at 4°C, washed 3 × in PBS. A 1: 400 dilution of anti-rabbit Alexa Fluor 405 antibody (Invitrogen, Carlsbad, CA, USA) in PBST was incubated for 1 hour at room temperature, and washed 3 × in PBS. Cells were then counterstained for actin with Alexa Fluor Phalloidin 568 (Invitrogen, Carlsbad, CA, USA) and nuclei with Sytox green (Invitrogen, Carlsbad, CA, USA) as per manufacturer's recommendations. Cells were imaged using a Leica TCS SP2 confocal microscope.

### Quantitative real time PCR experiments

The following procedures were performed as to comply with the MIQE guidelines [27].

### RNA extraction and cDNA synthesis

RNA was immediately extracted from duplicate wells of 3 separate cell cultures. RNA extraction and genomic DNA removal was performed using a RNeasy plus kit (Qiagen Inc., Chatsworth, CA, USA) as per manufacturer's recommendations. RNA was concentrated by ethanol precipitation and quantified using a NanaoDrop 1000 spectrophotometer (Thermo Fisher Scientific, Waltham, MA, USA). Only RNA with an A260/280 ratio between 1.8 and 2.1 and an A260/230 above 1.9 was used for cDNA synthesis. For samples where enough RNA was obtained (excludes day 2), the integrity of the RNA was confirmed by gel electrophoresis. Residual genomic DNA was removed using the genomic DNA wipeout buffer included in the Quantitect reverse transcription kit (Qiagen Inc., Chatsworth, CA, USA). 800 ng of RNA was reverse transcribed into cDNA for 30 min at 42°C using a Quantitect reverse transcription kit (Qiagen Inc., Chatsworth, CA, USA) as per manufacturer's recommendations.

### Quantitative PCR

qPCR was performed using a Stratagene MX3005P QPCR system (Stratagene, La Jolla, CA, USA) with Brilliant II SYBR (Stratagene, La Jolla, CA, USA). cDNA used in qPCR was first diluted 80-fold with nuclease free H_2_O. Each qPCR reaction mixture contained 7.5 μl 2 × Brilliant II SYBR green master mix (Surestart Taq DNA polymerase, 2.5 mM MgCl_2_), 6 μl cDNA (80-fold dilution), 500 nM each primer and RNase free water to a final volume of 15 μl. Amplification was performed in duplicate in 96 well plates (Stratagene, La Jolla, CA, USA) with the following thermal cycling conditions: initial activation 95°C for 10 minutes, followed by 40 cycles of 15 s at 95°C, 30 s at 60°C, and 30 s at 72°C. Control reactions included a no template control (NTC) and no reverse transcription control (-RT). Dissociation analysis of the PCR products was performed by running a gradient from 60 to 95°C to confirm the presence of a single PCR product. Products were also sequenced to confirm identity. A 4-fold dilution series made from known concentrations of plasmid containing the PCR inserts was used to calculate absolute copy numbers for each of the genes examined. PCR efficiencies for input into Bestkeeper were calculated from a dilution series (1/20, 1/40, 1/80, 1/160, 1/320, 1/640) of cDNA

Standards for calculating absolute copy number for each gene were prepared by cloning the PCR product from each primer pair into a T/A pCR4-TOPO vector (Invitrogen, Carlsbad, CA, USA) and transformation of chemically competent TOP10 *Escherichia coli *cells (Invitrogen,. Carlsbad, CA, USA). Individual colonies were grown and plasmids purified using Fastprep plasmid purification method (Eppendorf, Hamburg, Germany). The concentration of each plasmid was calculated based on absorbance at 260 nm, and a dilution series produced for calculation of copy number via qPCR.

### Primer design

Primers were designed using NetPrimer (Premier BioSoft, Palo Alto, CA, USA) to have Tm of 60°C, and where possible, were designed to cross an exon-exon junction to avoid amplification of contaminating genomic DNA. To determine exon-intron junction sites, genomic sequences for orthologous genes from *Danio rario*, *Gasterosteus aculeatus*, *Oryzias latipes*, *Takifugu rubripes *and *Tetraodon nigroviridis *were retrieved from Ensembl http://www.ensembl.org/index.html, and compared to the *Salmo salar *cDNA sequences using the Spidey software tool http://www.ncbi.nlm.nih.gov/spidey/. Primers were designed across conserved exon-intron junctions and used at a final concentration of 500 nM. *18SrRNA*, at 500 nM gave poor amplification efficiencies, however this was improved using a final concentration of 1.5 μM. The primers used for qPCR are listed in table 3 and have been submitted to rtprimerdb http://www.rtprimerdb.org/[28].

**Table 3:** qPCR primer sequences, and amplification parameters.

Gene	Primer sequence (5'-3')	Amplicon size (bp)	Tm (°C)	E (%) plasmid	R^2^ plasmid	E (%) cDNA	R^2^ cDNA
*EF1α*	f: GAATCGGCTATGCCTGGTGAC r: GGATGATGACCTGAGCGGTG	141	86.0	96.0	0.998	99.5	0.999
*RNApolII*	f: CCAATACATGACCAAATATGAAAGG r: ATGATGATGGGGATCTTCCTGC	157	84.8	95.3	0.996	98.5	0.999
*18SrRNA*	f: TCGGCGTCCAACTTCTTA r: GCAATCCCCAATCCCTATC	189	86.5	95.6	0.995	94.5	0.999
*Ppia*	f: CATCCCAGGTTTCATGTGC r: CCGTTCAGCCAGTCAGTGTT	203	85.9	96.4	0.998	96.5	0.999
*Pgk1*	f: CTCGGTGATGGGGCTTAGG r: TCATTGGTGGAGGCGACA	160	87.0	98.1	0.999	99.5	0.999
*Actb*	f: TGACCCAGATCATGTTTGAGACC r: CTCGTAGATGGGTACTGTGTGGG	146	83.8	93.2	0.997	100	0.999
*Hprt1*	f: CCGCCTCAAGAGCTACTGTAAT r: GTCTGGAACCTCAAACCCTATG	255	81.8	92.1	0.996	90.0	0.997
*Sdha*	f: CATGTTACCAAGGGCTGCAT r: GTGTCAGATGATATCTCAACCCAG	207	85.8	99.0	0.997	95.5	0.998
*Des*	f: GTCCATCTGGATCTGCACCT r: GGCTGCTTTCAGAGCTGATG	169	82.8	99.5	0.998	99.0	0.996

PCR efficiencies, amplicon melting temperature (Tm) and correlation coefficients of standard curves (from plasmid and cDNA) for candidate reference genes and desmin.

### Data analysis

The stability of candidate reference genes was determined using geNorm [15], Normfinder [17] and Bestkeeper [16]. Input data for geNorm and Normfinder were absolute values derived from a plasmid standard curve with the data for geNorm transformed as per author's guidelines. Input for Bestkeeper was the Cq values, and the PCR efficiencies calculated from a dilution series (1/20, 1/40, 1/80, 1/160, 1/320, 1/640) of cDNA. Normfinder Analysis of inter and intra group variation was performed on all data, days 2–11 and days 11–20. Statistical analysis was performed with Minitab (Minitab Inc).

## Abbreviations

DMEM: Dulbecco's modified eagle's media; PBS: phosphate buffered saline; Actb: actin beta; Ef1α: eukaryote elongation factor 1 alpha; RnapolII: Rna polymerase 2; Gapd: glyceraldehyde-3-phosphate dehydrogenase; Hprt1: hypoxanthine phosphoribosyl transferase 1; Ppia: prolylpeptidyl isomerase A; Pgk: phosphoglycerate kinase; Sdha: succinate dehydrogense; 18SrRNA: 18S ribosomal RNA; Des: desmin.

## Authors' contributions

NB performed the experimental work and wrote the first draft of the manuscript. IJ contributed to study design and writing of the manuscript. Both authors read and approved the final manuscript.
